# Supplementary material for: Predicting visual function by interpreting a neuronal wiring diagram
Source: Nature. 2024 Oct 2;634(8032):113–23. doi: 10.1038/s41586-024-07953-5 (PMC11446822; doi:10.1038/s41586-024-07953-5)
Supplement: Supplementary file 6 — CRF and ERF predictions for individual TmY4 and TmY9 cells. Analogous to Supplementary Data 3, but for TmY target types. Shown are the top four monosynaptic pathways, the strongest pathway passing through each of the top ten intermediary types (ranking from Extended Data Fig. 7), and the trisynaptic pathway Tm1–TmY–Dm3–TmY (see the section entitled Prediction of spatial normalization). [file 41586_2024_7953_MOESM6_ESM.zip › DataS4/TmY9q/720575940641626075.pdf]

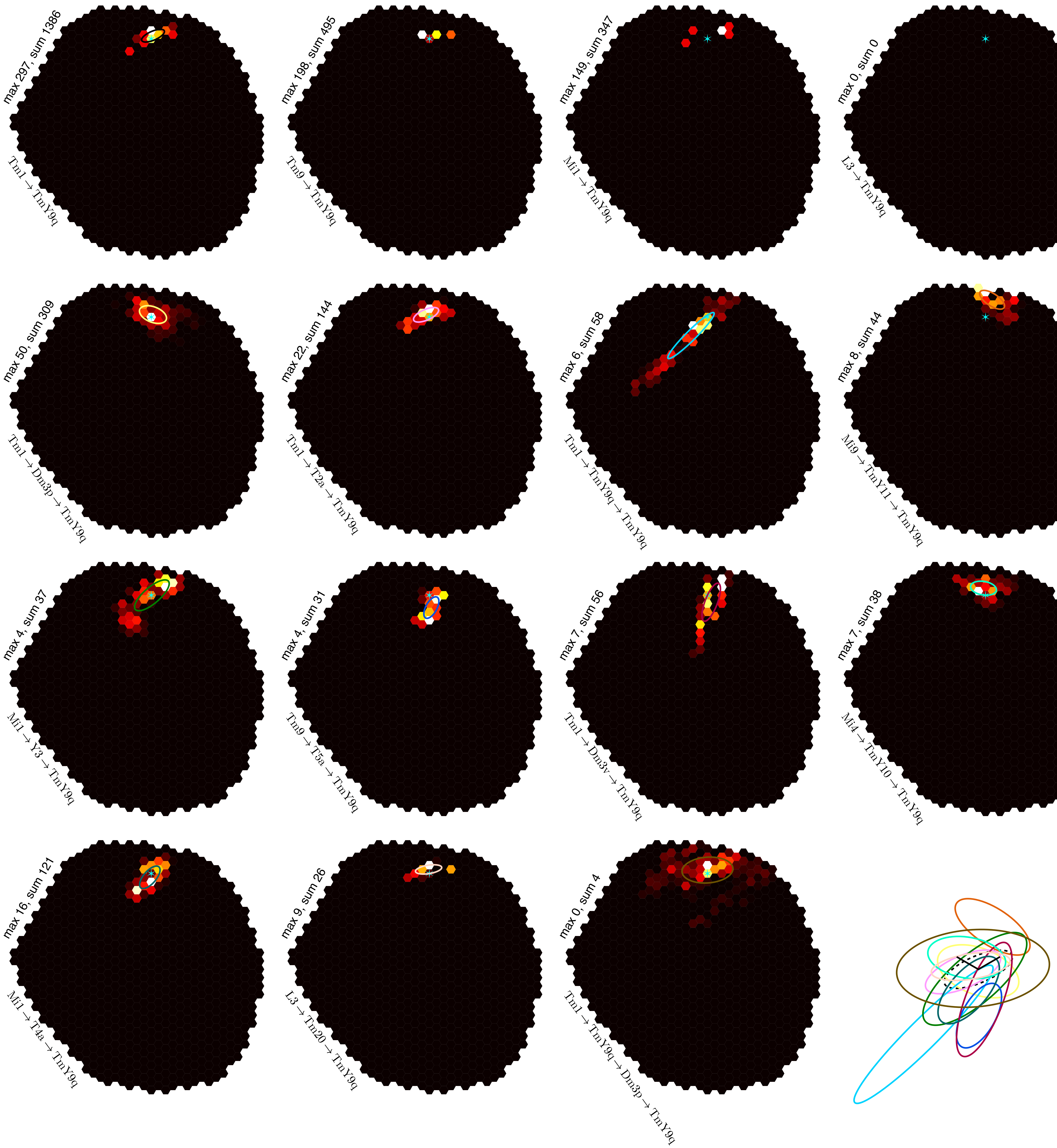
max 297, sum 1386
Tm1 → TmY9q
max 198, sum 495
Tm9 → TmY9q
max 149, sum 347
Mi1 → TmY9q
max 0, sum 0
L3 → TmY9q
max 50, sum 309
Tm1 → Dm3p → TmY9q
max 22, sum 144
Tm1 → T2a → TmY9q
max 6, sum 58
Tm1 → TmY9q → TmY9q
max 8, sum 44
Mi9 → TmY11 → TmY9q
max 4, sum 37
Mi1 → Y3 → TmY9q
max 4, sum 31
Tm9 → T5a → TmY9q
max 7, sum 56
Tm1 → Dm3v → TmY9q
max 7, sum 38
Mi4 → TmY10 → TmY9q
max 16, sum 121
Mi1 → T4a → TmY9q
max 9, sum 26
L3 → Tm20 → TmY9q
max 0, sum 4
Tm1 → TmY9q → Dm3p → TmY9q
